# Supplementary material for: Integrated Metabolomic and Gut Microbiome Profiles Reveal Postmortem Biomarkers of Fatal Anaphylaxis
Source: Int J Mol Sci. 2025 Jun 29;26(13):6292. doi: 10.3390/ijms26136292 (PMC12249648; doi:10.3390/ijms26136292)
Supplement: Supplementary file 1 [file ijms-26-06292-s001.zip › ijms-3643510-supplementary.pdf]

Supplementary Figures

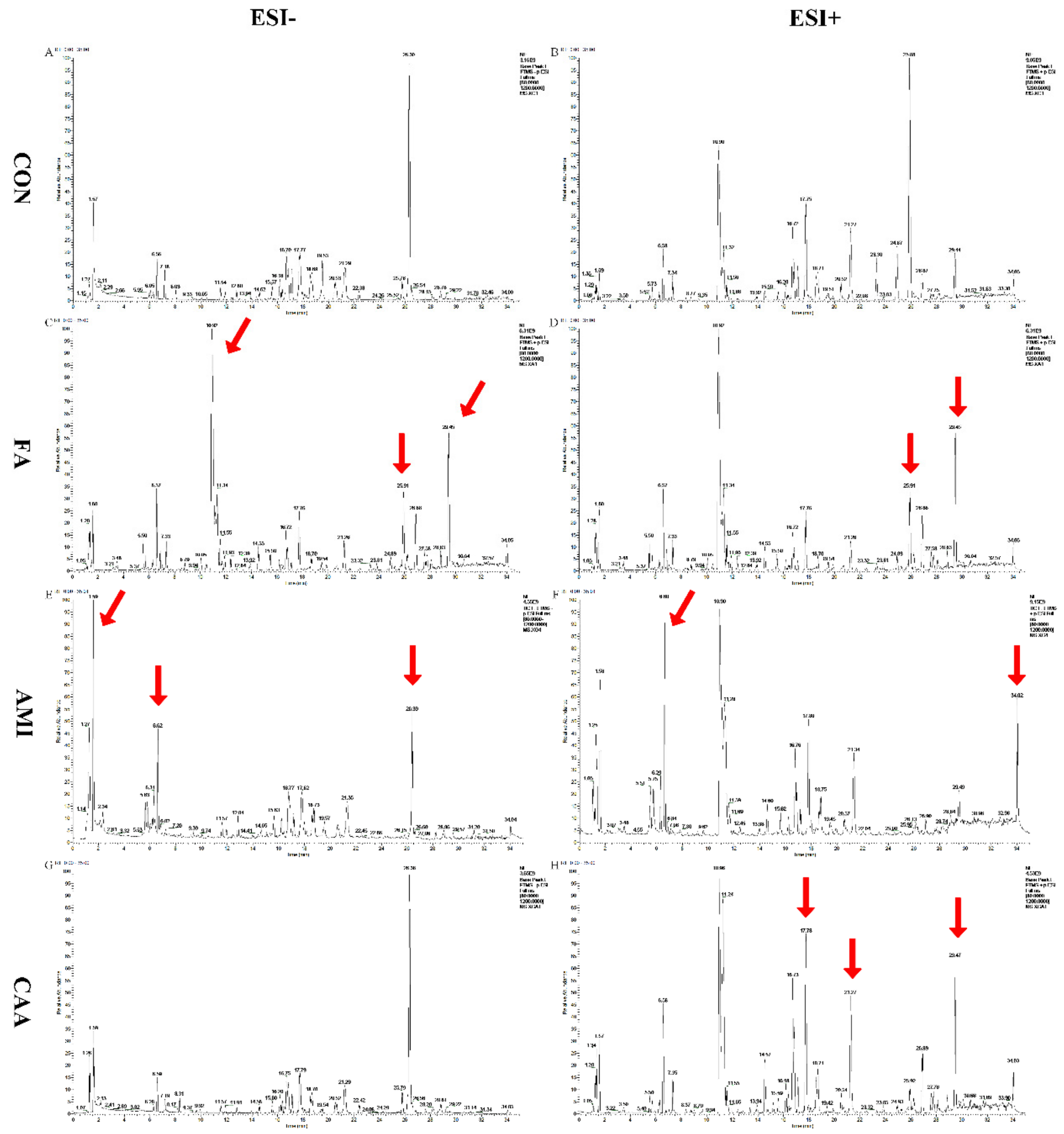

Figure S1. The typical total ion chromatograms of representative samples. The arrows show some representative peaks.

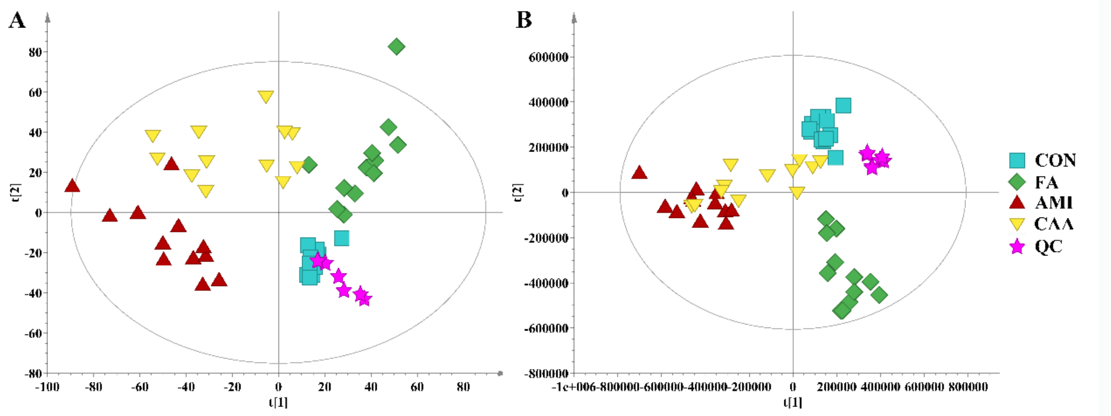

Figure S2. PCA scatter plot (A) and PLS-DA scatter plot (B) of overall samples.

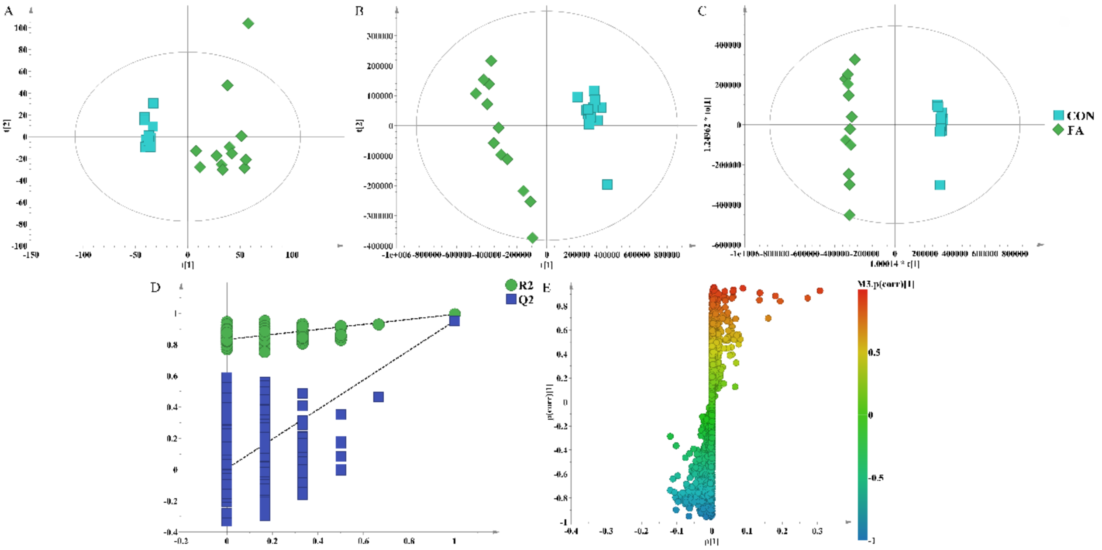

Figure S3. PCA scatter plot (A), PLS-DA scatter plot (B), OPLS-DA scatter plot (C), PLS-DA model validation plot (D), and S-plot (E) of the fatal anaphylaxis group with the control group.

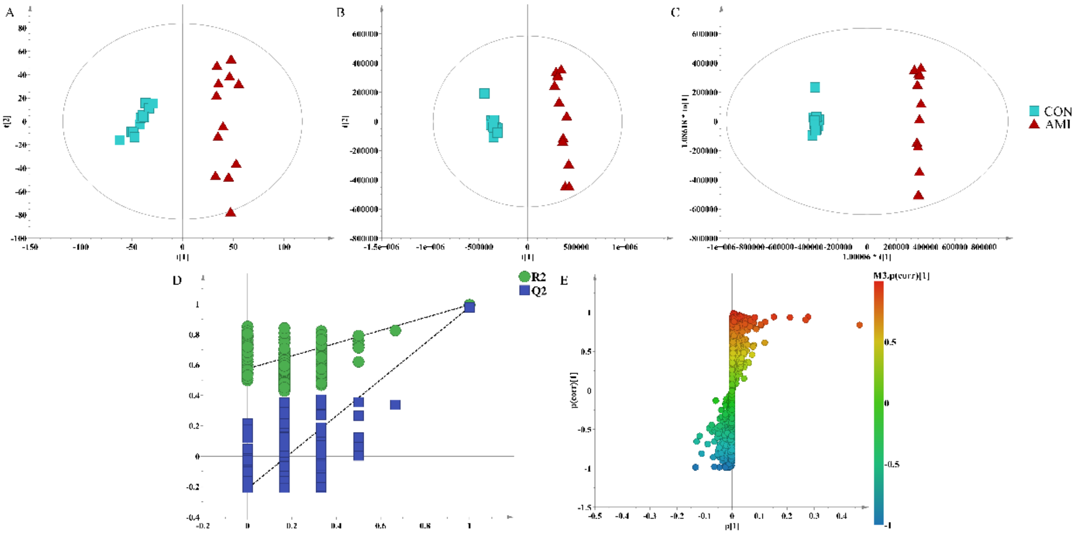

Figure S4. PCA scatter plot (A), PLS-DA scatter plot (B), OPLS-DA scatter plot (C), PLS-DA model validation plot (D) and S-plot (E) of acute myocardial infarction compared with control group.

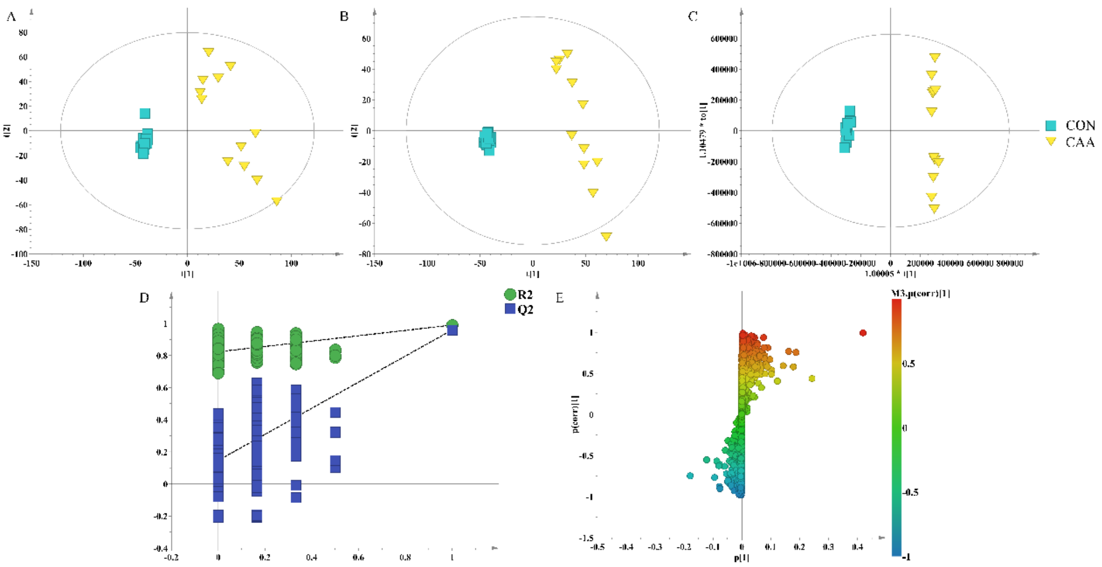

Figure S5. PCA scatter plot (A), PLS-DA scatter plot (B), OPLS-DA scatter plot (C), PLS-DA model validation plot (D) and S-plot (E) of coronary atherosclerosis with anaphylaxis compared with control group.

Supplementary Tables

Table S1. Differential metabolites in plasma of fatal anaphylaxis

Number	Metabolites	VIP value	*P* value	Molecular formula	Mass charge ratio（m/z）	Retention time（min）	Trend	ESI mode
1	Betaine	8.33300	1.23E-04	$C_5H_{11}NO_2$	117.07906	1.301	↑	+
2	D-(+)-Proline	8.01240	2.17E-05	$C_5H_9NO_2$	115.06344	1.376	↑	+
3	Hippuric acid	7.90090	3.80E-07	$C_9H_9NO_3$	179.05780	6.203	↑	−
4	Cholic acid	7.72628	5.29E-06	$C_{24}H_{40}O_5$	408.28651	11.934	↑	−
5	Taurochenodeoxycholic Acid (sodium salt)	6.25208	1.06E-04	$C_{26}H_{45}NO_6S$	499.29700	8.200	↑	−
6	L-Phenylalanine	5.86781	1.83E-06	$C_9H_{11}NO_2$	165.07884	5.530	↑	+
7	3-Indoxyl sulphate	5.76434	2.41E-04	$C_8H_7NO_4S$	213.00902	6.042	↑	−
8	Taurochenodeoxycholic acid	5.51510	7.48E-04	$C_{26}H_{45}NO_6S$	499.29687	10.005	↑	−
9	Arachidonic acid	5.34687	2.16E-03	$C_{20}H_{32}O_2$	304.23937	26.244	↑	−
10	Citric acid	5.06846	5.18E-05	$C_6H_8O_7$	192.02675	1.598	↑	−
11	Gluconic acid	4.97891	6.62E-09	$C_6H_{12}O_7$	196.05803	1.293	↑	−
12	D-(+)-Malic acid	4.75742	5.54E-05	$C_4H_6O_5$	134.02113	1.574	↑	−
13	Phenylacetylglycine	4.75283	3.69E-03	$C_{10}H_{11}NO_3$	193.07348	6.436	↑	−
14	L-Norleucine	4.65802	1.14E-06	$C_6H_{13}NO_2$	131.09462	3.469	↑	+
15	Palmitoyl sphingomyelin	4.64138	8.87E-07	$C_{39}H_{79}N_2O_6P$	702.56626	28.228	↓	+
16	L-Glutamic acid	4.12397	2.60E-05	$C_5H_9NO_4$	147.05289	1.287	↑	−
17	Valine	4.10738	3.30E-05	$C_5H_{11}NO_2$	117.07906	1.590	↑	+
18	Erucamide	3.66898	2.53E-04	$C_{22}H_{43}NO$	337.33410	32.011	↑	+
19	D-Erythro-sphingosine 1-phosphate	3.61774	6.46E-04	$C_{18}H_{38}NO_5P$	379.24823	14.384	↓	+

20	Taurine	3.54316	9.12E-06	$C_2H_7NO_3S$	125.01431	1.270	↑	−
21	DL-Arginine	3.10925	1.75E-06	$C_6H_{14}N_4O_2$	174.11151	1.251	↓	+
22	3-Hydroxybutyric acid	2.89250	4.32E-05	$C_4H_8O_3$	104.04712	1.654	↑	−
23	DL-Glutamine	2.85234	8.84E-04	$C_5H_{10}N_2O_3$	146.06896	1.268	↑	+
24	Spermidine	2.84960	4.59E-04	$C_7H_{19}N_3$	145.15775	1.087	↑	+
25	6-Hydroxycaproic acid	2.81758	2.38E-04	$C_6H_{12}O_3$	132.07839	6.486	↑	−
26	DL-Lysine	2.69982	2.55E-03	$C_6H_{14}N_2O_2$	146.10539	1.114	↑	+
27	L-Isoleucine	2.68380	1.55E-04	$C_6H_{13}NO_2$	131.09463	3.186	↑	+
28	D-(+)-Pyroglutamic Acid	2.63673	6.94E-06	$C_5H_7NO_3$	129.04254	1.271	↑	+
29	Indole	2.62335	2.61E-07	C_8H_7N	117.05793	6.204	↑	+
30	Succinic acid	2.61284	1.41E-05	$C_4H_6O_4$	118.02599	2.967	↑	−
31	Methylmalonic acid	2.61035	1.41E-05	$C_4H_6O_4$	118.02643	2.966	↑	−
32	Acetylcholine	2.44185	6.14E-05	$C_7H_{15}NO_2$	145.11014	1.382	↑	+
33	Trigonelline	2.31883	1.67E-03	$C_7H_7NO_2$	137.04757	1.354	↑	+
34	Deoxycholic Acid	2.26229	6.22E-04	$C_{24}H_{40}O_4$	392.29134	12.412	↑	−
35	D-Sphingosine	2.15403	2.55E-03	$C_{18}H_{37}NO_2$	299.28215	14.844	↑	+
36	L-Threonicacid	2.12022	1.29E-04	$C_4H_8O_5$	136.03670	1.321	↑	−
37	D-(+)-Pipecolinic acid	2.05901	1.82E-03	$C_6H_{11}NO_2$	129.07892	1.114	↑	+
38	Propionylcarnitine	1.96850	7.51E-06	$C_{10}H_{19}NO_4$	217.13111	1.600	↑	+
39	L-(+)-Citrulline	1.74106	1.90E-03	$C_6H_{13}N_3O_3$	175.09543	1.288	↑	+
40	Methylimidazoleaceticacid	1.62370	1.10E-05	$C_6H_8N_2O_2$	140.05848	1.465	↑	+
41	Stearic Acid	1.47833	2.35E-04	$C_{18}H_{36}O_2$	284.27163	31.122	↑	−
42	L-Kynurenine	1.47711	3.83E-03	$C_{10}H_{12}N_2O_3$	208.08453	5.498	↑	+
43	Fumaricacid	1.42320	1.58E-04	$C_4H_4O_4$	116.01042	1.589	↑	−
44	Nicotinamide	1.31151	4.91E-04	$C_6H_6N_2O$	122.04802	2.280	↑	+
45	Imidazoleaceticacid	1.21564	1.85E-03	$C_5H_6N_2O_2$	126.04289	1.406	↑	+

46	Dodecanedioicacid	1.21442	3.40E-04	$C_{12}H_{22}O_4$	230.15126	9.831	↑	−
47	Acetyl-L-carnitine	1.21044	9.84E-04	$C_9H_{17}NO_4$	203.11564	2.143	↑	+
48	2-Methylhippuricacid	1.14115	3.11E-03	$C_{10}H_{11}NO_3$	193.07333	6.848	↑	−
49	α,α-Trehalose	1.03096	2.49E-03	$C_{12}H_{22}O_{11}$	342.11597	1.400	↑	−
50	Myristyl sulfate	1.00587	4.63E-03	$C_{14}H_{30}O_4S$	294.18586	21.239	↑	−

"↑" or "↓" represents that metabolites were significantly up-regulated or down-regulated in the fatal anaphylaxis group compared with the control group. "+" or "−" represents a positive or negative ion scan mode. The highlighted 13 metabolites were present in both fatal anaphylaxis and acute myocardial infarction groups.

Table S2. Differential metabolites in plasma of acute myocardial infarction

**Number**	**Metabolites**	**VIP value**	***P* value**	**Molecular formula**	**Mass charge ratio （m/z）**	**Retention time （min）**	**Trend**	**ESI mode**
1	2-Amino-1,3,4-octadecanetriol	16.37510	1.46E-10	$C_{18}H_{39}NO_3$	317.29274	11.206	↑	+
2	Citric acid	11.76200	8.54E-09	$C_6H_8O_7$	192.02675	1.598	↑	−
3	Docosahexaenoic acid	5.38849	1.00E-07	$C_{22}H_{32}O_2$	328.23929	25.808	↓	−
4	D-(+)-Proline	5.23420	4.65E-03	$C_5H_9NO_2$	115.06344	1.376	↑	+
5	L-Norleucine	4.35929	1.57E-03	$C_6H_{13}NO_2$	131.09462	3.469	↑	+
6	Cholic acid	3.88688	8.87E-04	$C_{24}H_{40}O_5$	408.28651	11.934	↑	−
7	L-Phenylalanine	3.87070	1.28E-03	$C_9H_{11}NO_2$	165.07884	5.530	↑	+
8	Valine	3.76023	3.61E-03	$C_5H_{11}NO_2$	117.07906	1.590	↑	+
9	Palmitoyl sphingomyelin	3.75505	1.85E-06	$C_{39}H_{79}N_2O_6P$	702.56626	28.228	↓	+
10	valpromide	3.58339	6.55E-08	$C_8H_{17}NO$	143.13096	9.153	↑	+
11	Isobutyric acid	3.55549	5.19E-12	$C_4H_8O_2$	88.05275	7.360	↓	+
12	L-Isoleucine	3.32754	4.01E-03	$C_6H_{13}NO_2$	131.09463	3.186	↑	+
13	Mono(2-ethylhexyl) phthalate (MEHP)	3.07314	7.35E-04	$C_{16}H_{22}O_4$	278.15149	20.693	↑	+

14	Linoleic acid	2.85791	9.24E-04	$C_{18}H_{32}O_2$	280.23948	26.578	↓	−
15	Caprolactam	2.66523	2.02E-09	$C_6H_{11}NO$	113.08420	5.938	↓	+
16	DL-Malic acid	2.50645	2.39E-03	$C_4H_6O_5$	134.02162	1.574	↑	−
17	L-Tyrosine	2.27850	4.56E-03	$C_9H_{11}NO_3$	181.07375	3.060	↑	+
18	Choline	2.27660	1.49E-04	$C_5H_{13}NO$	103.09995	1.258	↓	+
19	L-(+)-Citrulline	2.26451	2.63E-03	$C_6H_{13}N_3O_3$	175.09543	1.288	↑	+
20	3-Hydroxybutyric acid	2.21308	3.41E-04	$C_4H_8O_3$	104.04712	1.654	↑	−
21	6-Hydroxycaproic acid	2.11801	4.74E-04	$C_6H_{12}O_3$	132.07839	6.486	↑	−
22	Hexadecanamide	2.00235	3.51E-03	$C_{16}H_{33}NO$	255.25587	25.714	↓	+
23	Taurochenodeoxycholic Acid (sodium salt)	1.72024	2.89E-03	$C_{26}H_{45}NO_6S$	499.29678	10.073	↑	−
24	4-phenolsulfonic acid	1.70925	2.26E-03	$C_6H_6O_4S$	173.99817	5.780	↓	−
25	Bis(2-ethylhexyl)phthalate	1.52086	6.19E-05	$C_{24}H_{38}O_4$	390.27634	31.998	↓	+
26	1-Dodecyl-2-pyrrolidinone	1.45910	9.41E-07	$C_{16}H_{31}NO$	253.24016	11.685	↑	+
27	DL-Alanine	1.38554	4.56E-03	$C_3H_7NO_2$	89.04799	1.263	↑	+
28	Triethanolamine	1.37744	1.79E-08	$C_6H_{15}NO_3$	149.10507	1.389	↑	+
29	Linoleoyl Ethanolamide	1.32639	1.59E-04	$C_{20}H_{37}NO_2$	323.28207	21.760	↓	+
30	Stearic Acid	1.07452	1.52E-04	$C_{18}H_{36}O_2$	284.27163	31.122	↑	−
31	Decanamide	1.03449	3.62E-05	$C_{10}H_{21}NO$	171.16213	12.572	↑	+
32	Glycocholicacid	1.01844	5.66E-06	$C_{26}H_{43}NO_6$	465.30863	12.615	↓	−

"↑" or "↓" represents that metabolites were significantly up-regulated or down-regulated in the fatal anaphylaxis group compared with the control group. "+" or "−" represents a positive or negative ion scan mode. The highlighted 13 metabolites were present in both fatal anaphylaxis and acute myocardial infarction groups.

Table S3. Differential metabolites in plasma of coronary atherosclerosis with anaphylaxis

Number	Metabolites	VIP value	*P* value	Molecular formula	Mass charge ratio （m/z）	Retention time （min）	Trend	ESI mode
1	2-Amino-1,3,4-octadecanetriol	12.7268	4.38E-03	$C_{18}H_{39}NO_3$	317.29274	11.206	↑	+
2	Citric acid	8.10268	6.53E-03	$C_6H_8O_7$	192.02675	1.598	↑	−
3	Cholic acid	6.16104	3.05E-04	$C_{24}H_{40}O_5$	408.28651	11.934	↑	−
4	L-(+)-Lactic acid	6.11283	1.87E-03	$C_3H_6O_3$	90.03123	2.219	↓	−
5	Gluconic acid	5.64407	3.40E-08	$C_6H_{12}O_7$	196.05803	1.293	↑	−
6	L-(-)-Carnitine	5.21408	1.67E-05	$C_7H_{15}NO_3$	161.10504	1.301	↑	+
7	D-(+)-Proline	4.43778	4.74E-04	$C_5H_9NO_2$	115.06344	1.376	↑	+
8	L-Phenylalanine	4.27551	5.65E-06	$C_9H_{11}NO_2$	165.07884	5.53	↑	+
9	L-Norleucine	4.13917	3.15E-07	$C_6H_{13}NO_2$	131.09462	3.469	↑	+
10	Taurochenodeoxycholic acid	4.0889	1.54E-04	$C_{26}H_{45}NO_6S$	499.29687	10.005	↑	−
11	Linoleic acid	3.77426	7.61E-03	$C_{18}H_{32}O_2$	280.24031	26.584	↑	−
12	Cytosine	3.59377	1.74E-05	$C_4H_5N_3O$	111.04338	1.589	↓	+
13	Methylmalonic acid	3.34624	2.61E-08	$C_4H_6O_4$	118.02643	2.966	↑	−
14	Docosahexaenoic acid	3.30261	2.17E-02	$C_{22}H_{32}O_2$	328.23929	25.808	↓	−
15	Oleic acid	3.28444	1.78E-03	$C_{18}H_{34}O_2$	282.25524	28.832	↑	−
16	DL-Malic acid	3.27108	2.69E-08	$C_4H_6O_5$	134.02162	1.574	↑	−
17	Taurochenodeoxycholic Acid (sodium salt)	3.26018	3.51E-03	$C_{26}H_{45}NO_6S$	499.297	8.2	↑	−
18	Glycocholic acid	3.23439	8.60E-03	$C_{26}H_{43}NO_6$	465.30852	9.727	↑	−
19	Tridemorph	3.17949	6.00E-05	$C_{19}H_{39}NO$	297.30285	29.767	↑	+
20	3-Indoxyl sulphate	3.07227	1.53E-02	$C_8H_7NO_4S$	213.00902	6.042	↓	−
21	valpromide	2.72651	4.99E-03	$C_8H_{17}NO$	143.13096	9.153	↑	+

22	trans-3-Indoleacrylic acid	2.70473	6.08E-03	$C_{11}H_9NO_2$	187.06321	5.746	↓	+
23	Valine	2.68424	7.81E-04	$C_5H_{11}NO_2$	117.07906	1.59	↑	+
24	D-(+)-Tryptophan	2.6147	2.65E-03	$C_{11}H_{12}N_2O_2$	204.08975	5.746	↓	+
25	Mono(2-ethylhexyl) phthalate (MEHP)	2.61236	1.08E-02	$C_{16}H_{22}O_4$	278.15149	20.693	↑	+
26	Corticosterone	2.57716	1.24E-06	$C_{21}H_{30}O_4$	346.21413	9.674	↑	+
27	Isobutyric acid	2.57468	2.57E-02	$C_4H_8O_2$	88.05275	7.36	↓	+
28	DL-Stachydrine	2.53773	9.79E-04	$C_7H_{13}NO_2$	143.09454	1.428	↓	+
29	L-Isoleucine	2.44807	1.53E-04	$C_6H_{13}NO_2$	131.09463	3.186	↑	+
30	Palmitoyl sphingomyelin	2.43232	1.78E-02	$C_{39}H_{79}N_2O_6P$	702.56626	28.228	↓	+
31	D-(+)-Glucose	2.29792	7.01E-04	$C_6H_{12}O_6$	180.06315	1.314	↑	−
32	L-Glutamic acid	2.29115	3.58E-04	$C_5H_9NO_4$	147.05289	1.287	↑	−
33	Trigonelline	2.28395	1.20E-04	$C_7H_7NO_2$	137.04757	1.354	↑	+
34	Caprolactam	2.2637	6.51E-03	$C_6H_{11}NO$	113.0842	5.938	↓	+
35	Propionylcarnitine	2.15584	3.65E-05	$C_{10}H_{19}NO_4$	217.13111	1.6	↑	+
36	6-Hydroxycaproic acid	2.08108	1.74E-06	$C_6H_{12}O_3$	132.07839	6.486	↑	−
37	L-Ascorbic acid 2-sulfate	2.07554	1.74E-02	$C_6H_8O_9S$	255.98823	1.595	↑	−
38	Methyl indole-3-acetate	2.04526	1.34E-02	$C_{11}H_{11}NO_2$	189.0788	8.395	↑	+
39	D-(+)-Malic acid	2.04334	4.40E-05	$C_4H_6O_5$	134.02113	1.574	↑	−
40	Hexadecanamide	2.01272	4.32E-03	$C_{16}H_{33}NO$	255.25587	25.714	↑	+
41	D-(+)-Pyroglutamic Acid	1.96729	7.56E-05	$C_5H_7NO_3$	129.04254	1.271	↑	+
42	DL-Glutamine	1.9478	1.16E-03	$C_5H_{10}N_2O_3$	146.06896	1.268	↑	+
43	Palmitoylcarnitine	1.81611	8.13E-03	$C_{23}H_{45}NO_4$	399.33448	17.934	↑	+
44	Stearic Acid	1.78346	4.11E-08	$C_{18}H_{36}O_2$	284.27163	31.122	↑	−
45	(±)9-HpODE	1.76964	6.01E-07	$C_{18}H_{32}O_4$	312.22967	17.859	↑	−
46	Taurine	1.75846	3.98E-05	$C_2H_7NO_3S$	125.01431	1.27	↑	−

47	Erucamide	1.74462	3.76E-02	$C_{22}H_{43}NO$	337.3341	32.011	↑	−
48	L-Tyrosine	1.72506	2.64E-04	$C_9H_{11}NO_3$	181.07375	3.06	↑	+
49	Isoquinoline	1.65095	1.34E-02	C_9H_7N	129.05785	8.394	↑	+
50	Palmitic acid	1.62472	1.08E-02	$C_{16}H_{32}O_2$	256.23972	28.212	↑	−
51	D-Sphingosine	1.58551	1.39E-03	$C_{18}H_{37}NO_2$	299.28215	14.844	↑	+
52	Imidazoleaceticacid	1.52151	1.61E-05	$C_5H_6N_2O_2$	126.04289	1.406	↑	+
53	DL-Arginine	1.48622	3.44E-02	$C_6H_{14}N_4O_2$	174.11151	1.251	↓	+
54	L-(+)-Citrulline	1.39772	9.80E-04	$C_6H_{13}N_3O_3$	175.09543	1.288	↑	+
55	DL-Alanine	1.36198	1.68E-05	$C_3H_7NO_2$	89.04799	1.263	↑	+
56	Linoleoyl Ethanolamide	1.35303	2.55E-03	$C_{20}H_{37}NO_2$	323.28207	21.76	↑	+
57	Glycochenodeoxycholic Acid (sodium salt)	1.34737	9.42E-03	$C_{26}H_{43}NO_5$	449.31354	12.184	↑	−
58	Hexanoylcarnitine	1.26603	1.67E-05	$C_{13}H_{25}NO_4$	259.17812	6.477	↑	+
59	(±)9(10)-DiHOME	1.21711	1.85E-03	$C_{18}H_{34}O_4$	314.24505	14.908	↑	−
60	1-Dodecyl-2-pyrrolidinone	1.20718	7.44E-03	$C_{16}H_{31}NO$	253.24016	11.685	↑	+
61	L-Kynurenine	1.1806	6.77E-10	$C_{10}H_{12}N_2O_3$	208.08445	1.607	↓	+
62	Bis(2-ethylhexyl)phthalate	1.1749	1.28E-02	$C_{24}H_{38}O_4$	390.27634	31.998	↓	+
63	Palmitoleic Acid	1.16926	1.24E-02	$C_{16}H_{30}O_2$	254.22399	25.547	↑	−
64	Nicotinamide	1.12352	3.05E-05	$C_6H_6N_2O$	122.04802	2.28	↑	+
65	Phenylacetylglycine	1.11583	2.50E-02	$C_{10}H_{11}NO_3$	193.07348	6.436	↓	−
66	Cuminaldehyde	1.09523	4.15E-05	$C_{10}H_{12}O$	148.08864	16.254	↑	+
67	2,3-dinor Prostaglandin E1	1.0918	9.79E-05	$C_{18}H_{30}O_5$	326.20926	8.675	↑	−
68	Myristyl sulfate	1.09032	9.11E-03	$C_{14}H_{30}O_4S$	294.18586	21.239	↑	−
69	Deoxycholic Acid	1.06043	6.26E-03	$C_{24}H_{40}O_4$	392.29036	16.029	↓	−
70	Acetyl-L-carnitine	1.03233	1.57E-02	$C_9H_{17}NO_4$	203.11564	2.143	↑	+
71	Dodecanedioicacid	1.01979	5.93E-05	$C_{12}H_{22}O_4$	230.15126	9.831	↑	−

"↑" or "↓" represents that metabolites were significantly up-regulated or down-regulated in the coronary atherosclerosis with anaphylaxis group compared with the control group. "+" or "–" represents a positive or negative ion scan mode. The 12 metabolites highlighted in yellow are present in both the FA and AMI groups, the 22 metabolites highlighted in green are present in the FA group, and the 14 metabolites highlighted in blue are present in the AMI group.

Table S4. The identified differential gut microbiota by LEfSe analysis

Biomarker names	Log value	Group	LDA value	*P* value
p_Firmicutes.c_Clostridia.o_Peptostreptococcales_Tissierellales.f_Peptostreptococcaceae.g_Romboutsia	8.56	FA	8.24	3.48E-02
p_Actinobacteriota.c_Actinobacteria.o_Corynebacteriales.f_Corynebacteriaceae.g_Corynebacterium	8.48	FA	8.11	1.43E-02
p_Bacteroidota.c_Bacteroidia.o_Bacteroidales.f_Prevotellaceae.g_Alloprevotella	8.29	FA	8.01	2.54E-02
p_Firmicutes.c_Clostridia.o_Oscillospirales.f_Oscillospiraceae.g_NK4A214_group	8.32	FA	7.95	1.26E-02
p_Bacteroidota.c_Bacteroidia.o_Bacteroidales.f_Bacteroidaceae.g_Bacteroides	8.27	FA	7.89	5.40E-05
p_Bacteroidota.c_Bacteroidia.o_Bacteroidales.f_Prevotellaceae.g_Prevotella	8.07	FA	7.65	2.03E-02
p_Firmicutes.c_Clostridia.o_Oscillospirales.f_Ruminococcaceae.g_Eubacterium_siraeum_group	8.02	FA	7.59	4.74E-02
p_Firmicutes.c_Clostridia.o_Lachnospirales.f_Lachnospiraceae.g_Lachnospiraceae_NK4A136_group	7.94	FA	7.55	4.77E-02
p_Firmicutes.c_Clostridia.o_Lachnospirales.f_Lachnospiraceae.g_Blautia	7.91	FA	7.53	6.57E-03
p_Verrucomicrobiota.c_Verrucomicrobiae.o_Verrucomicrobiales.f_Akkermansiaceae.g_Akkermansia	7.69	FA	7.41	3.96E-02
p_Firmicutes.c_Clostridia.o_Clostridiales.f_Clostridiaceae.g_Clostridium_sensu_stricto_1	7.74	FA	7.40	3.38E-03
p_Firmicutes.c_Clostridia.o_Lachnospirales.f_Lachnospiraceae.g_Roseburia	7.72	FA	7.34	5.85E-03
p_Firmicutes.c_Clostridia.o_Oscillospirales.f_Oscillospiraceae.g_Colidextribacter	7.68	FA	7.31	5.98E-03
p_Firmicutes.c_Clostridia.o_Oscillospirales.f_Oscillospiraceae.g_Oscillibacter	7.72	FA	7.30	1.40E-02
p_Firmicutes.c_Clostridia.o_Oscillospirales.f_Ruminococcaceae.g_Fournierella	7.51	FA	7.15	1.53E-02
p_Firmicutes.c_Clostridia.o_Peptococcales.f_Peptococcaceae.g_Peptococcus	7.42	FA	7.10	3.54E-03
p_Firmicutes.c_Clostridia.o_Oscillospirales.f_Clostridium_methylpentosum_group.g_Clostridium_methylpentosum_group	5.68	FA	7.07	2.03E-02
p_Firmicutes.c_Clostridia.o_Oscillospirales.f_Ruminococcaceae.g_Pygmaiobacter	7.37	FA	7.00	4.71E-02
p_Bacteroidota.c_Bacteroidia.o_Bacteroidales.f_Prevotellaceae.g_Prevotellaceae_Ga6A1_group	8.63	AMI	8.31	2.03E-02
p_Desulfobacterota.c_Desulfovibrionia.o_Desulfovibrionales.f_Desulfovibrionaceae.g_Desulfovibrio	7.89	AMI	7.55	2.75E-03
p_Firmicutes.c_Clostridia.o_Oscillospirales.f_Butyricicoccaceae.g_UCG_008	7.85	AMI	7.51	2.03E-02
p_Firmicutes.c_Bacilli.o_Staphylococcales.f_Staphylococcaceae.g_Staphylococcus	6.40	AMI	6.85	2.51E-02
p_Firmicutes.c_Clostridia.o_Lachnospirales.f_Lachnospiraceae.g_Eubacterium_hallii_group	7.09	AMI	6.83	2.03E-02
p_Actinobacteriota.c_Actinobacteria.o_Corynebacteriales.f_Dietziaceae.g_Dietzia	6.89	AMI	6.64	2.49E-02
p_Firmicutes.c_Clostridia.o_Lachnospirales.f_Lachnospiraceae.g_Eubacterium_ruminantium_group	8.60	CAA	8.22	9.59E-03
p_Firmicutes.c_Clostridia.o_Lachnospirales.f_Lachnospiraceae.g_Tuzzerella	7.50	CAA	7.24	1.67E-02
p_Firmicutes.c_Clostridia.o_Oscillospirales.f_Oscillospiraceae.g_UCG_005	7.22	CAA	7.01	3.87E-02
p_Proteobacteria.c_Gammaproteobacteria.o_Pseudomonadales.f_Moraxellaceae.g_Psychrobacter	7.24	CAA	6.95	4.15E-02

Table S5. The candidate biomarkers with AUC > 0.7

No.	**Candidate biomarker**	**AUC**	***P* value**
1	2,3-dinor Prostaglandin E1	0.9618	2.57E-04
2	Imidazoleaceticacid	0.9566	1.79E-09
3	Taurochenodeoxycholic Acid (sodium salt)	0.9566	4.10E-06
4	D-Sphingosine	0.9531	7.89E-07
5	Taurochenodeoxycholic acid	0.9444	1.91E-06
6	Taurine	0.9219	2.22E-06
7	Cuminaldehyde	0.9219	7.48E-06
8	Dodecanedioicacid	0.9097	5.03E-06
9	Methylimidazoleaceticacid	0.8941	1.71E-05
10	Gluconic acid	0.8837	8.82E-09
11	D-(+)-Malic acid	0.8785	1.67E-04
12	Choline	0.8785	6.60E-05
13	DL-Arginine	0.8750	1.94E-06
14	Methylmalonic acid	0.8681	9.19E-06
15	Stearic Acid	0.8663	3.41E-06
16	Trigonelline	0.8646	7.62E-07
17	Propionylcarnitine	0.8628	4.40E-04
18	Fumaricacid	0.8594	6.19E-04
19	Corticosterone	0.8594	8.70E-07
20	Palmitic acid	0.8576	5.19E-06
21	L-Ascorbic acid 2-sulfate	0.8542	7.80E-06
22	Cholic acid	0.8524	1.76E-06
23	9(10)-DiHOME	0.8507	6.02E-05
24	L-Glutamic acid	0.8403	9.44E-05
25	D-(+)-Pyroglutamic Acid	0.8385	4.95E-05
26	Deoxycholic Acid	0.8351	6.25E-03
27	Hexanoylcarnitine	0.8229	4.37E-03
28	Nicotinamide	0.8212	8.05E-04
29	Oleic acid	0.8142	2.27E-04
30	Palmitoylcarnitine	0.8073	2.82E-04
31	Acetylcholine	0.8003	1.10E-03
32	D-(+)-Tryptophan	0.7969	6.91E-04
33	trans-3-Indoleacrylic acid	0.7951	8.34E-04
34	DL-Alanine	0.7934	1.44E-02
35	Myristyl sulfate	0.7917	6.03E-04
36	L-Phenylalanine	0.7847	2.49E-04
37	D-(+)-Pipecolinic acid	0.7778	3.94E-03
38	Palmitoleic Acid	0.7760	1.35E-03
39	9-HpODE	0.7743	2.47E-04
40	Erucamide	0.7726	4.20E-04
41	4-phenolsulfonic acid	0.7691	5.76E-03

42	D-(+)-Proline	0.7517	8.18E-03
43	Linoleic acid	0.7465	1.79E-03
44	Betaine	0.7396	2.14E-03
45	Isobutyric acid	0.7378	2.32E-02
46	Triethanolamine	0.7344	6.22E-03
47	Spermidine	0.7309	1.93E-03
48	Hippuric acid	0.7292	8.46E-04
49	DL-Glutamine	0.7274	8.98E-03
50	6-Hydroxycaproic acid	0.7257	3.19E-02
51	L-Threonicacid	0.7188	1.63E-03
52	Indole;1-Benzazole	0.7170	5.30E-04
53	L-(+)-Lactic acid	0.7170	4.51E-03
54	DL-Malic acid	0.7153	1.95E-02
55	DL-Lysine	0.7083	1.06E-02
56	*g_Peptococcus*	0.9861	4.95E-04
57	*g_NK4A214_group*	0.9583	1.25E-04
58	*g_Tuzzerella*	0.9549	9.24E-04
59	*g_Alloprevotella*	0.9097	2.10E-02
60	*g_Eubacterium_ruminantium_group*	0.8958	5.40E-03
61	*g_Clostridium_sensu_stricto_1*	0.8611	1.71E-02
62	*g_Prevotellaceae_Ga6A1_group*	0.7917	3.00E-02
63	*g_UCG_008*	0.7500	2.24E-02
64	*g_Eubacterium_hallii_group*	0.7500	1.84E-02
65	*g_Blautia*	0.6667	4.24E-02
